# Illness cognition, illness perception and related factors in patients with lymphangioleiomyomatosis

**DOI:** 10.1186/s13023-025-03566-x

**Published:** 2025-02-19

**Authors:** Liting Huang, Lulu Yang, Ruoyun Ouyang, Siying Ren

**Affiliations:** 1 Department of Pulmonary and Critical Care Medicine, the Second Xiangya Hospital, Central South University, Changsha, 410011 Hunan China; 2 Unit of Respiratory Disease, Central South University, Changsha, 410011 Hunan China; 3 Clinical Medical Research Center for Pulmonary and Critical Care Medicine in Hunan Province, Changsha, 410011 Hunan Province China; 4 Diagnosis and Treatment Center of Respiratory Disease, Central South University, Changsha, 410011 Hunan China

**Keywords:** Patients, Illness cognition, Illness perception, Lymphangioleiomyomatosis, China

## Abstract

**Purpose:**

To explore the self-perceived illness cognition and perception status, as well as the relevant factors among lymphangioleiomyomatosis (LAM) patients.

**Methods:**

A web-based questionnaire survey was conducted in September 2023. A total of 121 LAM patients participated (including 16 patients with TSC-LAM), and the survey collected general demographic information, responses to a disease cognition questionnaire, and a simplified disease perception questionnaire.

**Results:**

LAM patients have a higher level of negative illness cognition and a lower level of positive illness cognition, specifically characterized by helplessness (15.74 ± 4.68 points), acceptance (16.00 ± 3.28 points), and perceived benefits (16.92 ± 3.86 points). Single-factor analysis of variance found significant correlations between cultural level, age, family average monthly income, use of rapamycin, use of home oxygen therapy, hospitalization frequency, disease duration, severity of respiratory distress, activity limitation, and the helplessness score of LAM patients (*p* ≤ 0.05); the number of children was significantly associated with acceptance scores of LAM patients (*p* ≤ 0.05); and whether surgery had been performed was significantly associated with acceptance and perceived benefits scores of LAM patients (*p* ≤ 0.05). Disease duration and activity limitation entered the regression equation for helplessness dimension, while whether surgery had been performed entered the regression equation for perceived benefits dimension, but no factor entered the regression equation for acceptance dimension. Applying the same analysis to disease perception, we found that the average score of the Illness Perception Questionnaire was 45.43 ± 8.97, with lower scores in the reverse-scored items of individual control, treatment, and understanding.

**Conclusions:**

LAM patients exhibit higher levels of helplessness, particularly among those with longer disease duration and greater activity limitations, leading to a more negative perception of the disease. Additionally, patients who have undergone surgical procedures tend to perceive fewer benefits. Furthermore, there is a significant correlation between illness perception and factors such as rapamycin usage, home oxygen therapy, disease duration and activity limitations caused by LAM. This indicates that clinical healthcare providers should pay more attention to LAM patients and their associated groups, providing both informational and psychological support.

## Introduction

Illness cognition and illness perception, refers to an individual’s cognitive assessment and emotional expression in the face of a disease state or health threat, thereby eliciting their psychological coping responses [1]. Research has shown that patients’ perception of their illness can influence their coping strategies and adjustments, such as healthcare-seeking behaviors, treatment adherence [2, 3], and emotional states, ultimately impacting the prognosis of the disease and the quality of life and social functioning of patients [4]. Positive disease cognition among cancer patients is identified as a significant predictor of their quality of life [5]. Currently, illness cognition has become an essential component of psychosocial support for cancer patients.

Lymphangioleiomyomatosis (LAM) as one of the first nationally recognized rare [6], is a multisystem, low-grade malignant neoplastic disease that can be classified into two types: sporadic LAM (S-LAM), which occurs without any known underlying genetic condition, and tuberous sclerosis complex-associated LAM (TSC-LAM), which is linked to mutations in the TSC1 or TSC2 genes [7]. It can lead to progressive pulmonary symptoms such as shortness of breath, cough, and pneumothorax [8]. LAM is also associated with the development of renal angiomyolipomas and chylous effusions [9]. The disease significantly impacts the physiological, psychological, and social health of patients. Psychological issues in LAM patients are closely related to their disease cognition and perception. Current treatments for LAM include the use of rapamycin, which has shown effectiveness in reducing the progression of the disease, as well as other supportive treatments such as oxygen therapy and surgical interventions for managing complications. However, there is currently a lack of research on the disease cognition and perception of LAM patients, including their levels of positive and negative disease cognition and perception. This study aims to investigate the illness cognition and perception levels of LAM patients, analyze the factors influencing their illness cognition and perception, and provide guidance for the clinical development of relevant interventions and support programs, thereby promoting the psychological well-being of LAM patients.

## Methods

### Sample and procedure

In September 2023, we conducted one cross-sectional survey online using the Questionnaire Star mini-program to explore the disease manifestations and related factors among patients with LAM. A total of 121 diagnosed LAM patients was recruited through online questionnaires (including 16 patients with TSC-LAM). The LAM patients’ inclusion criteria were (1) having a confirmed LAM diagnosis by according to the diagnostic criteria proposed by the Official American Thoracic Society/Japanese Respiratory Society [10]; (2) being aged 18 and over; and (3) being able to read and communicate in Mandarin. Participants with severe mental disorders (e.g., schizophrenia or intellectual disability), other malignancies, or severe physical conditions (such as stroke) were excluded. It is worth noting that the exclusion criteria did not specifically rule out participants with other systemic diseases, such as rheumatic or cardiovascular conditions. This decision was made to better reflect the real-world circumstances of LAM patients, many of whom may live with comorbid conditions. However, the potential influence of these systemic diseases on illness cognition and perception was not systematically assessed in this study, which has been acknowledged as a limitation in the Discussion section.

The participants were informed that declining to complete the survey would not impact their access to medical services. Written informed consents have been obtained from all participants prior to the survey. The study received approval from the Ethics Committee of the Second Xiangya Hospital of Central South University (LYEC2024-0141).

### Measurements

#### Sociodemographic and clinical characteristics

Sociodemographic information (including gender, residence, education, age, marital status, number of children, monthly household income, payment of medical expenses and economic burden) and clinical characteristics (history of pneumothorax, history of chylous effusions, whether renal angiomyolipoma, whether taking rapamycin, whether home oxygen therapy, number of hospitalizations, whether surgical treatment, whether diagnosis confirmed by biopsy, disease duration, current degree of dyspnea activity limitation from LAM and symptoms present most of the time in the past year) were collected.

#### The illness cognition questionnaire(ICQ)([11]

The ICQ is utilized to assess patients’ cognitive understanding of the stress and aversion characteristics associated with their illness. It evaluates patients’ perceptions of their illness from both positive and negative perspectives, encompassing three dimensions: helplessness, acceptance, and perceived benefits, totaling 18 items. The Likert 4-point scoring method is employed, with each dimension containing 6 items. Scores for each dimension range from 6 to 24 points. The Chinese version of the ICQ maintains consistency with the original questionnaire in terms of items and dimensions. The Cronbach’s α coefficient ranges from 0.855 to 0.878 [12].

#### The brief illness perception questionnaire(BIPQ)([13]

The BIPQ is used to assess patients’ cognitive and emotional representations of their illness, as well as their understanding of the disease, including nine items: consequences, timeline, personal control, treatment control, identity, coherence, concern, emotional response, and causes. The first eight items are scored from 0 to 10, with reverse scoring applied to items 3, 4, and 7. The total score ranges from 0 to 80, with higher scores indicating greater perceived severity of the disease. The ninth item is an open-ended question where patients list three factors they consider most important in causing the illness. The Chinese version of B-IPQ demonstrates good reliability and validity among breast cancer patients, with a Cronbach’s alpha coefficient of 0.77 [14].

### Statistical analysis

Data statistical analysis was performed using IBM SPSS Statistics Version 26.0. Continuous data were expressed as mean ± standard deviation (x ± s), while categorical data were presented as percentages. Analysis of variance (ANOVA) was utilized to explore the relationship between general data and dimensions of illness cognition and illness perception. Multiple regression analysis and partial correlation analysis were employed to identify factors influencing illness cognition and illness perception levels. Additionally, independent samples T-test was utilized to analyze differences between two groups.

## Results

### Patient survey

**Fig. 1:**
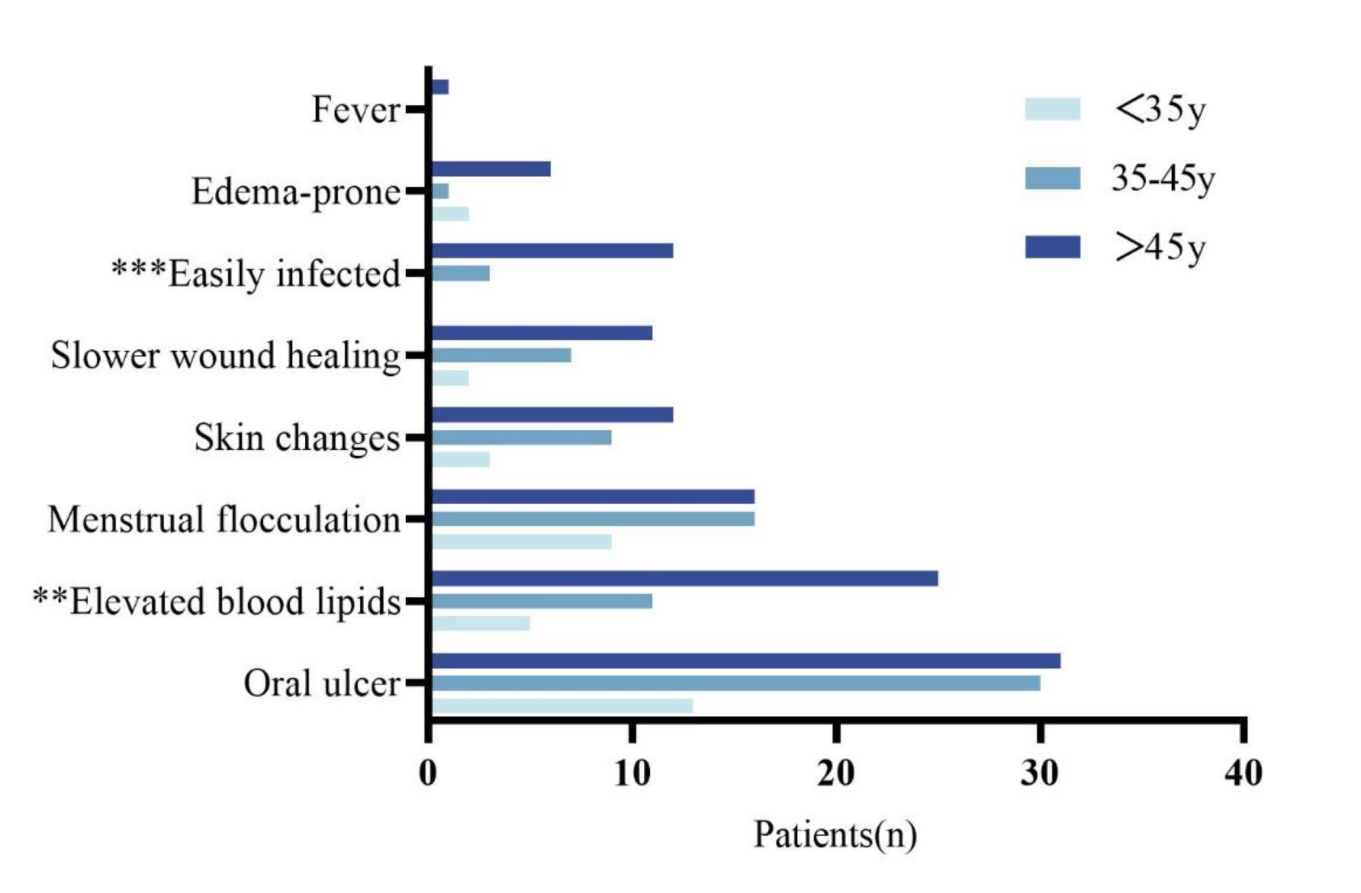
Side effects in patients with LAM who took rapamycin at different ages(*n* = 99). **P ≤ 0.01, ***P ≤ 0.001

Characteristics of the 121 LAM patients are presented in Table 1. All patients were female, and 82.6% were over 35 years old. A total of 33.9% of LAM patients had an education level up to secondary school. Among the patients, 78.5% had children. Approximately 41.3% of patients had a monthly income of no more than 3000 RMB. Rapamycin was taken by 81.8% of patients, most of whom experienced adverse reactions such as oral ulcers, elevated blood lipids, and menstrual irregularities. Significant differences in adverse reactions, such as elevated blood lipids and susceptibility to infections, were observed among patients of different age groups (*p* ≤ 0.01) (Fig. 1). Additionally, 41.3% of patients required home oxygen therapy, and 46.3% underwent surgical procedures. Furthermore, 37.2% of patients had a disease duration of more than 7 years.

**Table 1:** Demographic and clinical data on the 121 LAM patients

Variable	*n*( % )
Gender, female	121(100)
Residence	
City	68(56.2)
Country	53(42.8)
Education	
Up to secondary school	41(33.9)
College *	31(25.6)
Undergraduate	42(34.7)
Postgraduate and higher	7(5.8)
Age groups	
<35y	21(17.4)
35-45y	51(42.1)
>45y	49(40.5)
Marital status	
Married	102(84.3)
Divorced or widowed	19(15.7)
Number of children	
0	26(21.5)
1	71(58.7)
≥ 2	24(19.8)
Monthly household income	
< 3 000 RMB¥	50(41.3)
3000–5000 RMB¥	35(28.9)
5000–10,000 RMB¥	26(21.5)
>10,000 RMB¥	10(8.3)
Payment of medical expenses	
Partial reimbursement	97(80.2)
Fully reimbursed	4(3.3)
All self-funded	20(26.5)
Economic burden	
None	19(25.7)
Mildly	6(5.0)
Moderately	51(42.1)
Severe	45(37.2)
History of pneumothorax	
Yes	66(54.5)
No	55(45.5)
History of chylous effusions	
Yes	23(19.0)
No	98(81.0)
Renal angiomyolipoma	
Yes	36(29.8)
No	85(70.2)
Taking rapamycin	
Yes	99(81.8)
No	22(18.2)
Home oxygen therapy	
Yes	50(41.3)
No	71(58.7)
Number of hospitalizations	
0	8(6.6)
1–3	81(66.9)
4–5	10(8.3)
6–7	2(1.7)
>7	20(16.5)
Surgical treatment	
Yes	56(46.3)
No	65(53.7)
Diagnosis confirmed by biopsy	
Yes	53(43.8)
No	68(56.2)
Disease duration	
<1y	8(6.6)
1-3y	32(26.4)
3-5y	15(12.4)
5-7y	21(17.4)
8-10y	45(37.2)
Current degree of dyspnea	
None of these	24(19.8)
I only get breathless after strenuous exercise	8(6.6)
I get breathless when hurrying on level ground or walking up a slight incline	55(45.5)
I walk slower than people my own age	10(8.3)
I have to stop for breath after walking a few minutes on level ground	23(19.0)
I am too breathless to leave the house	1(0.8)
Activity limitation from LAM	
Not limit your activity	42(34.7)
Limit activities other than working	20(16.5)
Limit amount or type of work you can do	30(24.8)
Keep you from working	29(24.0)

* Three-year college or vocational school

### Severity and health care use

A total of 80.2% of LAM patients reported experiencing dyspnea on most days (Table 1). Activity limitation was severe, with 65.3% patients reporting work limitation or inability to work because of LAM. Nearly all patients reported being hospitalized for LAM in the past year, and 26.5% described to have been hospitalized more than three times.

### Present symptoms

**Fig. 2:**
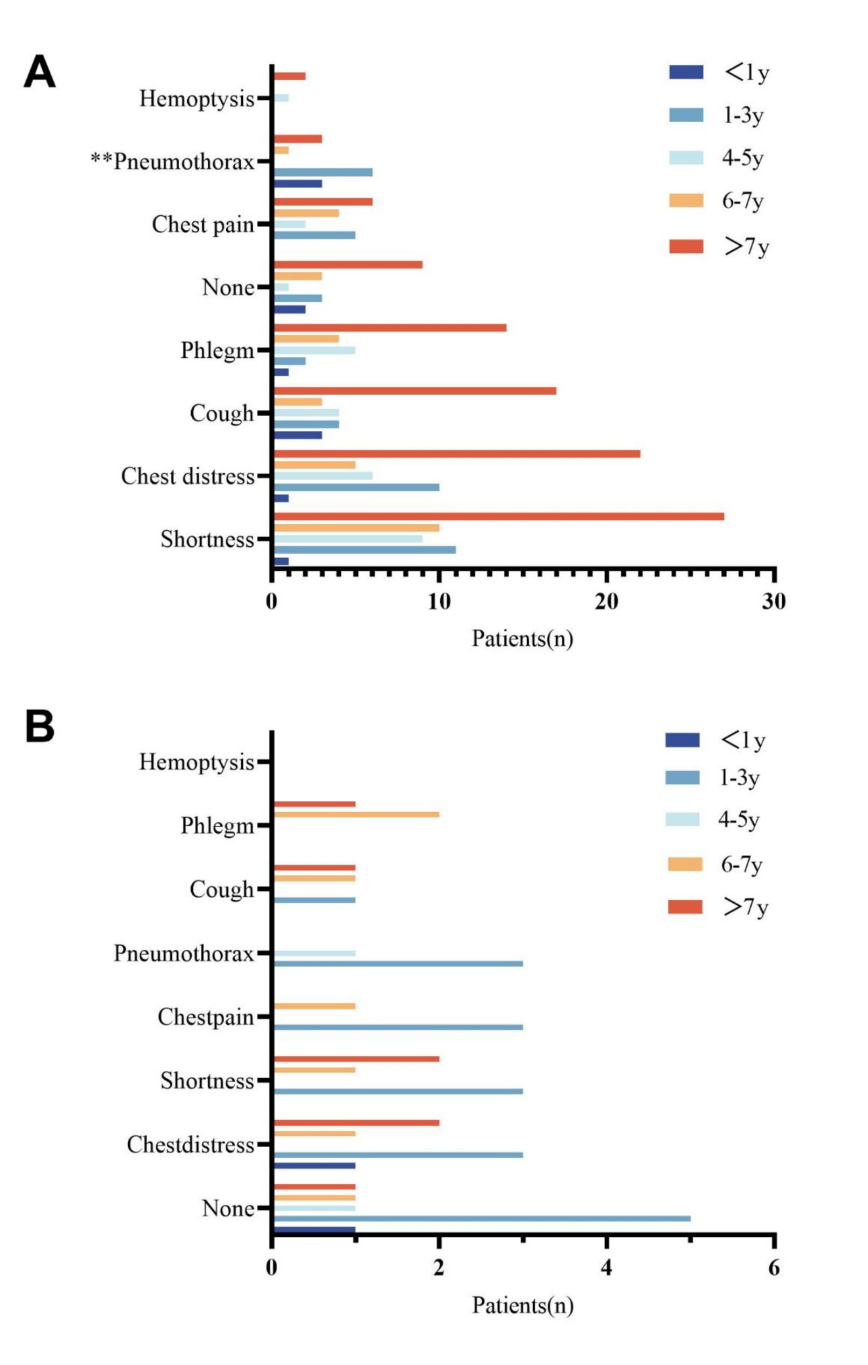
Present symptoms most of the time in the past year in 121 LAM patients with different disease durations: (**A**) taking rapamycin; (**B**) not taking rapamycin. ** p ≤ 0.01

The present symptoms most of the time in the past year in 121 LAM patients (99 patients take rapamycin, 22 patients do not take rapamycin) with different disease courses that divided into five courses are illustrated in Fig. 2. In patients taking rapamycin (Fig. 2A), symptoms mostly include shortness of breath, chest tightness, and cough, with hemoptysis being rare. Among patients with a disease duration exceeding 7 years, all symptoms except pneumothorax are most commonly observed, and there is a significant difference in the incidence of pneumothorax symptoms. And patients with a disease duration of more than 7 years are also the most common among those with no symptoms. In contrast, in patients not taking rapamycin (Fig. 2B), most do not exhibit any symptoms, or they experience shortness of breath and chest tightness, with no cases of hemoptysis. Additionally, patients with a disease duration of 1–3 years tend to have more discomfort symptoms.

### Illness cognition

The scores for the three dimensions of the ICQ were as follows: helplessness (15.74 ± 4.68) points; acceptance (16.00 ± 3.28) points; perceived benefits (16.92 ± 3.86) points. Single-factor analysis of variance (Table 2) revealed significant correlations between educational level, age grope, monthly household income, rapamycin use, home oxygen therapy, number of hospitalizations, disease duration, current degree of dyspnea, activity limitation from LAM and patients’ sense of helplessness in LAM (*p* ≤ 0.05). The number of children was significantly correlated with patients’ acceptance scores (*p* ≤ *0.05*). Whether surgery had been performed was significantly correlated with patients’ acceptance and perceived benefits levels (*p* ≤ 0.05).

**Table 2:** Results of univariate analysis of the illness cognition questionnaire (ICQ) subscale scores and related factors in the 121 LAM patients (only statistically significant results are shown)

Variable	Helpless			Acceptance			Perceived benefits		
	x ± s	t/F	*p*	x ± s	t/F	*p*	x ± s	t/F	*p*
Education		2.68	**0.05**		1.75	0.16		0.14	0.94
Up to secondary school	16.56 ± 4.85			15.37 ± 3.33			16.76 ± 3.78		
College *	16.65 ± 4.37			16.45 ± 3.38			17.26 ± 3.16		
Undergraduate	14.86 ± 4.46			15.93 ± 3.13			16.79 ± 4.31		
Postgraduate and higher	12.29 ± 4.61			18.14 ± 2.67			17.29 ± 5.03		
Age groups		5.57	**0.00**		1.02	0.37		1.17	0.32
<35y	14.90 ± 3.88			15.76 ± 3.19			16.38 ± 4.50		
35-45y	14.49 ± 4.02			15.61 ± 2,79			16.53 ± 2.62		
>45y	17.41 ± 5.18			16.51 ± 3.75			17.57 ± 3.80		
Number of children		1.32	0.27		3.39	**0.04**		1.04	0.36
0	14.73 ± 3.82			15.50 ± 2.97			16.15 ± 4.18		
1	16.31 ± 5.07			16.61 ± 3.50			17.34 ± 3.92		
≥ 2	15.17 ± 4.22			14.75 ± 2.47			16.54 ± 3.28		
Monthly household income		2.56	**0.05**		1.11	0.35		0.14	0.94
< 3 000 RMB¥	16.90 ± 4.38			15.48 ± 3.10			16.94 ± 3.72		
3000–5000 RMB¥	15.69 ± 4.87			16.14 ± 3.68			16.91 ± 3.54		
5000–10,000 RMB¥	14.50 ± 4.55			16.27 ± 2.96			16.65 ± 4.30		
>10,000 RMB¥	13.40 ± 4.77			17.40 ± 3.37			17.60 ± 4.93		
Taking rapamycin		5.92	**0.02**		0.08	0.78		0.16	0.69
Yes	16.22 ± 4.49			16.04 ± 3.29			16.86 ± 3.81		
No	13.59 ± 5.03			15.82 ± 3.28			17.23 ± 4.17		
Home oxygen therapy		27.21	**0.00**		0.54	0.47		0.26	0.61
Yes	18.14 ± 4.30			16.26 ± 3.62			17.14 ± 16.77		
No	14.06 ± 4.20			15.81 ± 3.02			16.77 ± 3.73		
Number of hospitalizations		4.11	**0.00**		1.36	0.25		0.40	0.81
0	17.13 ± 3.36			14.00 ± 3.12			16.63 ± 3.25		
1–3	14.64 ± 4.54			16.01 ± 3.09			16.80 ± 3.81		
4–5	19.20 ± 5.47			16.70 ± 3.83			17.70 ± 4.08		
6–7	16.00 ± 1.41			13.50 ± 0.71			14.50 ± 2.12		
>7	17.90 ± 4.01			16.65 ± 3.73			17.40 ± 4.47		
Surgical treatment		0.40	0.53		3.67	**0.05**		4.00	**0.04**
Yes	16.04 ± 4.60			15.39 ± 3.08			16.18 ± 3.25		
No	15.49 ± 4.77			16.52 ± 3.37			17.57 ± 4.24		
Disease duration		4.74	**0.00**		0.60	0.66		0.18	0.95
<1y	13.13 ± 2.95			15.50 ± 3.25			17.63 ± 3.02		
1-3y	13.63 ± 4.46			15.59 ± 3.43			16.66 ± 3.67		
3-5y	15.87 ± 4.44			16.87 ± 4.21			17.20 ± 4.13		
5-7y	15.71 ± 4.82			15.57 ± 2.82			16.57 ± 2.99		
>7y	17.69 ± 4.37			16.29 ± 3.07			17.07 ± 4.47		
Current degree of dyspnea		6.56	**0.00**		0.93	0.47		0.30	0.91
None of these	12.29 ± 3.65			15.50 ± 2.83			16.67 ± 3.70		
I only get breathless after strenuous exercise	14.88 ± 5.36			16.75 ± 3.62			18.13 ± 4.26		
I get breathless when hurrying on level ground or walking up a slight incline	16.09 ± 4.26			15.85 ± 3.24			16.73 ± 4.17		
I walk slower than people my own age	14.90 ± 4.48			14.90 ± 3.28			16.50 ± 3.34		
I have to stop for breath after walking a few minutes on level ground	18.87 ± 4.14			17.04 ± 3.69			17.39 ± 3.60		
I am too breathless to leave the house	23.00 ± 0.00			17.00 ± 0.00					
Activity limitation from LAM		16.23	**0.00**		1.27	0.29		1.07	0.36
Not limit your activity	12.81 ± 3.85			16.07 ± 3.32			16.93 ± 4.33		
Limit activities other than working	16.90 ± 4.67			16.30 ± 3.42			17.05 ± 4.07		
Limit amount or type of work you can do	15.57 ± 3.64			15.07 ± 2.78			16.00 ± 3.64		
Keep you from working	19.38 ± 4.01			16.66 ± 3.53			17.79 ± 3.13		

Factors with *p* ≤ 0.05 in the single-factor analysis were included in the multiple regression analysis (Table 3), with “entry” used for sub-variables and “stepwise entry” for other variables. Multiple regression analysis found that patients’ disease duration and activity restriction entered the regression equation for the helplessness dimension, while whether surgery had not been performed entered the regression equation for the perceived benefits dimension (*R*^*2*^*=*0.02). However, no factors entered the regression equation for the acceptance dimension.

**Table 3:** Results of multivariate regression analysis of the illness cognition questionnaire subscale scores and related factors in the 121 LAM patients( *n* = 121)

Subscale	Variable	Std.error	β	t	*p*	R^2^
Helplessness	Disease duration	0.31	0.43	5.53	**0.00**	0.24
	Activity limitation from LAM	0.26	0.27	3.45	**0.00**	0.31
Perceived benefits	Surgical treatment	0.70	0.18	2.00	**0.04**	0.03

Furthermore, factors with *p* ≤ 0.05 in the multiple regression analysis were subjected to partial correlation analysis (Table 4), revealing a significant correlation between patients’ disease duration, activity restriction (*p* ≤ 0.00), and their sense of helplessness in LAM illness cognition.

**Table 4:** Relationship of activity limitation from LAM and disease duration with the illness cognition questionnaire scores of the 121 LAM patients on the items of " helplessness “, " acceptance " and " perceived benefits” ( *n* = 121)

Variable	Disease duration			Activity limitation from LAM	
	*r*	*p*		*r*	*p*
Helplessness	0.30	**0.00**		0.45	**0.00**
Acceptance	0.07	0.46		0.00	1.00
Perceived benefits	-0.01	0.96		0.04	0.66

### Illness perception

The average score of the BIPQ is 45.43 ± 8.97, with consequences score of 5.97 ± 2.78; timeline score of 8.76 ± 2.24; personal control score of 4.12 ± 2.54; treatment control score of 3.20 ± 2.21; identity score of 6.72 ± 2.33; concern score of 7.98 ± 2.23; understanding score of 2.94 ± 2.15; and emotional response score of 5.73 ± 2.59.

Single-factor analysis of variance (Table 5) revealed significant correlations between patients’ age, family monthly income, economic burden, use of rapamycin, use of home oxygen therapy, disease duration, severity of respiratory distress, activity limitation, and consequences scores (*p* ≤ 0.05). Significant correlations were also found between patients’ age, use of rapamycin, disease duration, and timeline scores (*p* ≤ 0.05). Furthermore, disease duration, presence of chylous effusion, and treatment control scores showed significant correlations (*p* ≤ 0.05). Significant correlations between patients’ age, presence of chylous effusion, use of rapamycin, use of home oxygen therapy, disease duration, history of surgical treatment, severity of respiratory distress, activity limitation, and identity scores were observed (*p* ≤ 0.05). Moreover, the use of home oxygen therapy showed significant correlations with concern scores (*p* ≤ 0.05). Additionally, the presence of pneumothorax, use of home oxygen therapy, disease duration, and understanding scores showed significant correlations (*p* ≤ 0.05). Lastly, disease duration, presence of renal angiomyolipoma, use of rapamycin, use of home oxygen therapy, severity of respiratory distress, activity limitation, and emotional response scores were significantly correlated (*p* ≤ 0.05). No features were found to be related to personal control scores in the survey.

**Table 5:** Results of univariate analysis of the brief illness perception Questionnaire(BIPQ) subscale scores and related factors in the 121 LAM patients (only statistically significant results are shown)

Variable	Consequences	Timeline	Personal control	Treatment control	Identity	Concern	Understanding	Emotional response
Age groups								
<35y	5.10 ± 2.88	7.90 ± 3.03	4.52 ± 2.09	3.14 ± 2.33	5.67 ± 2.74	7.57 ± 2.42	3.48 ± 2.23	5.86 ± 3.02
35-45y	5.24 ± 2.35	8.60 ± 2.23	3.69 ± 2.28	3.22 ± 2.17	6.39 ± 2.01	7.61 ± 2.22	3.24 ± 2.14	5.35 ± 2.34
>45y	7.10 ± 2.82	9.29 ± 1.71	4.41 ± 2.92	3.20 ± 2.25	7.53 ± 2.24	8.55 ± 2.07	2.41 ± 2.15	6.06 ± 2.66
t/F	7.62***	3.10*	1.33	0.01	6.07**	2.75	2.72	0.96
Monthly household income								
< 3 000 RMB¥	6.84 ± 2.90	8.24 ± 2.69	4.48 ± 2.45	3.40 ± 2.30	6.88 ± 2.58	7.92 ± 2.28	2.96 ± 2.47	5.92 ± 2.82
3000–5000 RMB¥	5.77 ± 2.65	9.37 ± 1.37	3.69 ± 2.45	2.77 ± 2.10	6.86 ± 2.33	8.11 ± 2.03	2.86 ± 1.80	5.94 ± 2.66
5000–10,000 RMB¥	5.19 ± 2.64	8.69 ± 2.28	4.46 ± 2.92	3.77 ± 2.27	6.35 ± 1.90	7.81 ± 2.37	3.27 ± 2.11	4.96 ± 2.16
>10,000 RMB¥	4.30 ± 1.57	9.40 ± 1.58	3.00 ± 1.94	2.20 ± 1.55	6.50 ± 2.32	8.30 ± 2.54	2.30 ± 1.64	6.00 ± 2.16
t/F	3.81*	2.10	150	1.87	0.36	0.17	0.51	0.96
Economic burden								
None	4.67 ± 2.88	10.00 ± 0.00	4.67 ± 2.94	2.83 ± 1.47	8.17 ± 1.94	7.17 ± 2.79	2.83 ± 2.32	5.33 ± 2.25
Mildly	4.42 ± 1.84	8.53 ± 2.17	3.58 ± 3.08	2.84 ± 2.67	6.26 ± 1.63	9.00 ± 1.60	2.53 ± 1.95	5.05 ± 2.12
Moderately	5.88 ± 2.76	8.84 ± 2.15	4.20 ± 2.34	3.41 ± 1.87	6.49 ± 2.39	7.78 ± 2.11	3.08 ± 1.75	5.61 ± 2.68
Severe	6.89 ± 2.83	8.60 ± 2.50	4.20 ± 2.51	3.16 ± 2.47	7.00 ± 2.52	7.89 ± 2.44	2.98 ± 2.61	6.20 ± 2.70
t/F	4.39**	0.78	0.40	0.38	1.41	1.78	0.31	1.01
History of pneumothorax								
Yes	6.30 ± 2.69	8.70 ± 2.27	4.15 ± 2.41	3.05 ± 2.06	7.06 ± 2.28	7.73 ± 2.43	3.30 ± 2.31	5.68 ± 2.62
No	5.56 ± 2.87	8.84 ± 2.22	4.10 ± 2.70	3.38 ± 2.39	6.33v2.36	8,29 ± 1.94	2.51 ± 1.86	5.78 ± 2.59
t/F	2.14	0.12	0.02	0.69	3.01	1.93	4.22*	0.04
History of chylous effusions								
Yes	6.26 ± 2.49	8.96 ± 2.12	3.70 ± 2.51	2.26 ± 1.74	7.70 ± 2.01	7.96 ± 2.31	2.52 ± 2.23	5.70 ± 2.44
No	5.90 ± 2.86	8.71 ± 2.27	4.22 ± 2.55	3.42 ± 2.26	6.50 ± 2.36	7.99 ± 2.22	3.04 ± 2.12	5.73 ± 2.64
t/F	0.32	0.22	0.82	5.28*	5.05*	0.01	1.09	0.01
Renal angiomyolipoma								
Yes	6.19 ± 2.72	9.00 ± 2.12	4.31 ± 2.21	3.22 ± 1.76	6.97 ± 2.25	8.28 ± 2.05	2.72 ± 1.95	6.50 ± 2.68
No	5.87 ± 2.82	8.66 ± 2.29	4.05 ± 2.67	3.19 ± 2.39	6.62 ± 2.38	7.86 ± 2.30	3.04 ± 2.23	5.40 ± 2.50
t/F	0.34	0.59	0.26	0.01	0.56	0.89	0.54	4.68*
Taking rapamycin								
Yes	6.34 ± 2.65	8.97 ± 2.02	4.04 ± 2.56	3.08 ± 2.06	7.27 ± 1.99	7.96 ± 2.13	2.82 ± 2.08	6.07 ± 2.55
No	4.27 ± 2.81	7.82 ± 2.91	4.50 ± 2.48	3.73 ± 2.80	4.27 ± 2.23	8.09 ± 2.67	3.50 ± 2.41	4.18 ± 2.26
t/F	10.77***	4.91*	0.59	1.54	39.18****	0.06	1.83	10.28**
Home oxygen therapy								
Yes	4.04 ± 1.06	4.68 ± 0.71	3.14 ± 1.20	3.58 ± 0.95	4.10 ± 0.95	4.40 ± 0.86	4.02 ± 0.96	3.60 ± 1.12
No	2.64 ± 1.07	4.35 ± 1.11	3.31 ± 1.05	3.69 ± 0.99	3.30 ± 1.06	4.00 ± 1.03	3.59 ± 0.96	2.76 ± 1.18
t/F	49.71****	3.38	0.68	0.37	18.32****	5.08*	5.82*	15.48****
Surgical treatment								
Yes	6.32 ± 2.52	8.54 ± 2.37	4.50 ± 2.43	3.29 ± 2.12	7.21 ± 2.19	7.75 ± 2.41	3.09 ± 2.32	5.82 ± 2.64
No	5.66 ± 2.98	8.95 ± 2.12	3.80 ± 2.61	3.12 ± 2.30	6.31 ± 2.39	8.18 ± 2.05	2.82 ± 1.99	5.65 ± 2.57
t/F	1.70	1.05	2.31	0.16	4.68*	1.15	0.49	0.14
Disease duration								
<1y	4.88 ± 2.47	7.50 ± 2.88	2.75 ± 2.12	1.88 ± 1.88	5.25 ± 1.28	7.38 ± 2.56	3.13 ± 2.30	4.25 ± 2.25
1-3y	4.59 ± 2.30	8.63 ± 2.21	4.28 ± 2.26	3.09 ± 2.10	6.09 ± 2.45	8.31 ± 1.86	3.16 ± 2.02	5.06 ± 2.29
3-5y	6.53 ± 2.47	7.73 ± 2.60	2.87 ± 2.03	1.67 ± 1.59	7.53 ± 2.00	8.27 ± 1.91	3.67 ± 2.06	6.80 ± 2.48
5-7y	5.38 ± 3.20	8.19 ± 2.99	4.48 ± 2.58	4.24 ± 2.51	6.29 ± 2.59	6.81 ± 2.94	3.67 ± 2.74	4.76 ± 2.86
>7y	7.22 ± 2.52	9.69 ± 1.00	4.51 ± 2.79	3.53 ± 2.06	7.38 ± 2.15	8.31 ± 2.01	2.18 ± 1.74	6.56 ± 2.44
t/F	5.70****	4.11**	1.96	4.40**	3.10*	2.17	2.69*	4.06**
Current degree of dyspnea								
None of these	3.63 ± 2.00	7.96 ± 2.90	3.58 ± 1.69	2.75 ± 1.87	4.75 ± 2.01	7.67 ± 2.04	3.50 ± 1.41	4.50 ± 2.13
I only get breathless after strenuous exercise	4.63 ± 2.07	9.38 ± 1.77	3.13 ± 1.89	2.38 ± 1.85	5.88 ± 2.03	8.25 ± 2.76	2.63 ± 1.85	7.13 ± 3.14
I get breathless when hurrying on level ground or walking up a slight incline	6.05 ± 2.67	8.84 ± 2.19	4.13 ± 2.51	3.22 ± 2.23	7.11 ± 2.14	7.80 ± 2.36	2.98 ± 2.38	5.73 ± 2.50
I walk slower than people my own age	5.80 ± 2.35	8.60 ± 2.37	4.90 ± 3.07	4.30 ± 2.50	7.10 ± 2.28	8.00 ± 2.31	3.50 ± 2.27	4.20 ± 2.70
I have to stop for breath after walking a few minutes on level ground	8.65 ± 1.61	9.30 ± 1.52	4.78 ± 3.18	3.39 ± 2.48	7.96 ± 2.06	8.57 ± 1.93	2.17 ± 2.19	7.00 ± 2.15
I am too breathless to leave the house	8.00 ± 0.00	8.00 ± 0.00	2.00 ± 0.00	4.00 ± 0.00	8.00 ± 0.00	10.00 ± 0.00	2.00 ± 0.00	10.00 ± 0.00
t/F	11.88****	1.06	1.11	0.98	6.58****	0.66	1.13	4.44***
Activity limitation from LAM								
Not limit your activity	3.93 ± 2.05	8.57 ± 2.55	3.64 ± 2.01	2.91 ± 1.86	5.48 ± 2.12	7.83 ± 2.08	2.95 ± 1.82	4.90 ± 2.06
Limit activities other than working	5.40 ± 2.89	8.55 ± 2.84	3.80 ± 3.11	3.45 ± 2.89	6.65 ± 2.60	8.00 ± 2.81	2.90 ± 2.63	5.90 ± 2.95
Limit amount or type of work you can do	6.80 ± 2.11	8.80 ± 1.94	4.47 ± 2.36	3.20 ± 1.86	7.17 ± 2.02	7.47 ± 2.24	3.40 ± 1.79	5.73 ± 3.03
Keep you from working	8.45 ± 1.78	9.14 ± 1.55	4.69 ± 2.92	3.45 ± 2.53	8.14 ± 1.83	8.72 ± 1.87	2.48 ± 2.53	6.79 ± 2.24
t/F	26.99****	0.43	1.28	0.45	9.62****	1.70	0.90	3.24*

* p ≤ 0.05, ** p ≤ 0.01,*** p ≤ 0.001,**** p ≤ 0.0001

In single-factor analysis, factors with *p* ≤ 0.05 entered multiple regression analysis (Table 6), with “enter” used as a subvariable and other variables as “stepwise entry.” Multiple regression analysis found that patient disease duration, whether taking rapamycin, whether home oxygen therapy and activity limitation entered the “consequences” regression equation (*p* ≤ 0.01); disease duration entered the “timeline” regression equation(*P* ≤ *0.00*); whether having had chylous effusion and disease duration entered the “treatment control” regression equation (*p* ≤ 0.01; age, whether taking rapamycin, and activity limitation entered the “identity” regression equation (*p* ≤ 0.05); whether home oxygen therapy entered the “concern” regression equation (*p* ≤ 0.05); whether having had pneumothorax entered the “understanding” regression equation (*p* ≤ 0.05); whether taking rapamycin, whether home oxygen therapy and activity limitation entered the “emotional response” regression equation (*p* ≤ 0.05); however, no factors entered the “personal control” regression equation.

**Table 6:** Results of multivariate regression analysis of the brief illness perception Questionnaire subscale scores and related factors in the 121 LAM patients( *n* = 121)

Subscale	Variable	Std.error	β	t	*p*	R^2^
Consequences	Home oxygen therapy	-1.15	-0.21	-2.47	**0.00**	0.46
	Disease duration	0.14	0.18	2.56	**0.00**	0.50
	Current degree of dyspnea	0.17	0.25	3.04	**0.00**	0.49
	Activity limitation	0.19	0.46	5.74	**0.00**	0.40
Timeline	Disease duration	0.14	0.26	2.90	**0.00**	0.07
Treatment control	History of chylous effusions	0.49	0.22	2.54	**0.01**	0.09
	Disease duration	0.14	0.22	2.55	**0.01**	0.04
Identity	Age group	0.24	0.16	2.09	**0.04**	0.38
	Taking rapamycin	0.46	-0.39	-5.13	**0.00**	0.25
	Activity limitation	0.15	0.30	3.91	**0.00**	0.35
Concern	Home oxygen therapy	0.41	-0.20	-2.26	**0.03**	0.33
Understanding	History of pneumothorax	0.38	-0.21	-2.38	**0.00**	0.08
	Home oxygen therapy	0.38	0.26	2.91	**0.01**	0.05
Emotional response	Taking rapamycin	0.58	-0.22	-2.56	**0.00**	0.13
	Home oxygen therapy	0.46	-0.32	-3.63	**0.00**	0.09

Further, factors with *p* ≤ 0.05 from multiple regression analysis were entered into partial correlation analysis (Table 7), revealing that identity and emotional response scores were significantly correlated with whether taking rapamycin (*p* ≤ 0.05); consequences, identity, concern, understanding, and emotional response scores were significantly correlated with home oxygen therapy (*p* ≤ 0.05); consequences and treatment control scores were significantly correlated with disease duration (*p* ≤ 0.05); furthermore, consequences and identity scores were significantly correlated with activity limitation (*p* ≤ 0.05).

**Table 7:** Relationship of taking rapamycin, home oxygen therapy, disease duration and activity limitation with the brief illness perception questionnaire scores of the 121 LAM patients on the items of “consequences”, “timeline”, “treatment control”, “identity”, “concern”, “understanding” and “emotional response” (*n* = 121)

Variable	Taking rapamycin			Home oxygen therapy			Disease duration			Activity limitation	
	*r*	*p*		*r*	*p*		*r*	*p*		*r*	*p*
Consequences	-0.06	0.50		-0.57	**0.00**		0.19	**0.04**		0.46	**0.00**
Timeline	-0.12	0.19		-0.16	0.08		0.16	0.09		-0.02	0.87
Treatment control	0.17	0.07		-0.08	0.40		0.22	**0.02**		0.03	0.77
Identity	-0.37	**0.00**		-0.40	**0.00**		0.01	0.90		0.22	**0.02**
Concern	0.02	0.80		-0.20	**0.03**		0.01	0.91		0.11	0.25
Understanding	0.05	0.63		0.23	**0.01**		-0.08	0.41		0.05	0.57
Emotional response	-0.20	**0.03**		-0.32	**0.00**		0.14	0.15		0.15	0.12

## Discussion

This study found that all LAM patients surveyed were female, with an average age of 47.44 ± 8.62 years. This is because LAM is a rare lung disease that primarily affects women of childbearing age [15]. In the past year, patients who have been on rapamycin for more than 7 years exhibited the highest frequency of symptoms, except for pneumothorax. This might be because long-term use of rapamycin can reduce the risk of pneumothorax recurrence [16], and it also stabilizes the condition, leading to no symptoms. 65.3% of the LAM patients participating in this study had activity limitations due to the disease. Previous research has shown that although chronic disease patients must face long-term conditions that cannot be cured and result in limitations in daily functioning, chronic diseases are less life-threatening, but with the systematic and scientific management of chronic diseases, patients can have a correct and positive perception of and response to the disease [17].

We found that LAM patients have a higher level of negative perception of the disease, with a helplessness score of 15.74, while their positive perception of the disease is lower, with an acceptance score of 16.00 and a perceived benefit score of 16.92. The same as patients with other chronic diseases (such as breast cancer, prostate cancer, chronic fatigue, chronic pain, etc.), LAM patients exhibit higher levels of negative perception of the disease, with higher helplessness scores and lower acceptance and perceived benefit scores [18–20]. This may be related to the severity and prognosis of the disease.

Through single-factor analysis, we initially found significant correlations (*p* ≤ 0.05) between various factors and the level of helplessness among LAM patients, including educational level, age, monthly household income, rapamycin usage, use of home oxygen therapy, number of hospitalizations, disease duration, severity of respiratory distress, and activity limitations. Lower educational levels were associated with higher levels of helplessness. This finding aligns with studies on other pulmonary diseases, where educational level impacts patients’ psychological well-being [21]. Moreover, educational level may influence monthly family income, with lower incomes correlating with higher levels of helplessness. In cancers such as breast, ovarian, prostate, and lung cancers, income levels affect patients’ psychological health, and medical insurance can alleviate their burdens [22]. In LAM patients with 100% medical reimbursement, the average helplessness score was only 11.50 ± 1.73, and patients with no economic burden scored only 12.83 ± 5.91 for helplessness. Older age is associated with more days of poor mental health and higher levels of helplessness [23], as observed in gastric cancer as well [24]. Patients taking rapamycin showed higher levels of helplessness, and increased hospitalizations and severity of respiratory distress, as well as patients on home oxygen therapy, were associated with higher levels of helplessness, possibly related to disease severity and medication side effects [25, 26]. Subsequently, factors with *p* ≤ 0.05 from the single-factor analysis were subjected to multiple regression analysis and partial correlation analysis. It was found that disease duration and activity limitations were the main contributing factors (*p* ≤ 0.00). Longer disease duration and more activity limitations were associated with higher levels of helplessness. Similar to studies on breast cancer, treatment duration, which corresponds to disease duration, is correlated with patient depression [27]. Activity limitations may be influenced by the severity of the condition and the degree of respiratory distress.

Single-factor analysis revealed a significant correlation (*p* ≤ 0.05) between the number of children and the acceptance score of LAM patients. Additionally, whether a patient had undergone surgical procedures was significantly correlated (*p* ≤ 0.05) with both the acceptance and perceived benefits scores of LAM patients. However, the results of the multivariate analysis for both dimensions did not show significant differences. Patients with no children or only one child had higher acceptance scores compared to those with multiple children. Furthermore, patients with one child had higher acceptance scores than those with no children or multiple children. This may be related to the greater economic burden and life stress in families with multiple children, although children can bring hope to patients. Studies on breast cancer have shown that patients with children have significantly higher levels of hope compared to those without children [28]. LAM patients who underwent surgical procedures had lower acceptance and perceived benefits scores. Similar findings were observed in tongue cancer patients who underwent total or subtotal tongue resection, who were more prone to psychological disorders [29].

We found that the average score on the disease perception questionnaire was 45.43 ± 8.97. Scores were lower in the domains of personal control, treatment, and understanding, indicating that patients had lower perceived control over their disease, lacked confidence in medication management, and had limited understanding of the disease. As they lacked understanding of their condition, leading to psychological stress and influencing disease perception. In multiple linear regression analysis, only the regression equations for outcome and identity had *R*^*2*^>0.3, indicating good fit of the regression equations. Partial correlation analysis revealed significant correlations (*p* ≤ 0.05) between rapamycin usage, use of home oxygen therapy, disease duration, activity limitations caused by LAM, and patients’ disease perception. Similar findings have been observed in studies on various other types of cancer, although results regarding disease perception and perceived stress may vary depending on individual demographics [30–34].

This study recruited participants nationwide through an online questionnaire, shedding light on the cognitive and perceptual status of LAM patients regarding their disease. It has provided new insights into the factors influencing LAM patients’ awareness of their disease. However, our interpretation of the results is still constrained by several limitations. Firstly, due to the cross-sectional design, we cannot establish causal relationships. Recall and reporting biases in self-reported questionnaires may have influenced the results. Prospective longitudinal studies are needed to further establish causal relationships. Secondly, as LAM is a rare disease, the sample size is relatively small, and further recruitment of more patients is necessary to validate the findings. Thirdly, this study is not the first to report on the cognitive and perceptual status of LAM patients [35], but it is the first to analyze the key factors affecting patients’ psychological status, which include the use of rapamycin, use of home oxygen therapy, disease duration, and activity limitations caused by LAM. Fourthly, while this study did not exclude patients with systemic comorbidities, such as cardiovascular diseases or rheumatic diseases, we acknowledge that these comorbidities may influence the patients’ cognitive and perceptual status. The absence of detailed data on these comorbidities is a limitation of this study. Future research should systematically assess the effects of these comorbidities on illness cognition. Fifthly, we did not include data from the ninth item of the Brief Illness Perception Questionnaire (BIPQ), which asks patients to list the three most important factors they consider to have caused their illness. The responses to this open-ended question were highly variable and lacked structure, making it difficult to analyze and draw meaningful conclusions. Future studies could consider refining this type of data collection to ensure more systematic responses for analysis. Additionally, although we focused on disease-related factors, such as disease duration and activity limitations, other psychosocial factors (such as social support and coping strategies) could also play an important role in shaping illness perception and cognition. Future studies could explore these factors to gain a more comprehensive understanding of the psychological impact of LAM on patients.

## Conclusions

In summary, through the use of online questionnaires, we have gained initial insights into the level of understanding of LAM among patients. LAM patients exhibit high levels of helplessness, particularly among those with longer disease duration and greater activity limitations, leading to a more negative perception of the disease. Patients who have undergone surgical procedures perceive fewer benefits. Additionally, there are significant correlations between disease perception and factors such as disease duration, rapamycin usage, use of home oxygen therapy and activity limitations caused by LAM. This underscores the importance of healthcare workers focusing on individuals with longer disease duration and activity limitations due to the disease. Timely detection of psychological issues and targeted provision of information and emotional support are crucial for helping patients understand their condition and cope positively.

## Data Availability

The dataset used in this research and analysis was available from the corresponding author.

## Abbreviations

LAM: Lymphangioleiomyomatosis
ICQ: The Illness Cognition Questionnaire
BIPQ: The Brief Illness Perception Questionnaire
